# Polymorphic EBV-positive post-transplant lymphoproliferative disorder of the colon mimicking EBV-positive mucocutaneous ulcer: a case report

**DOI:** 10.1007/s12308-026-00712-7

**Published:** 2026-06-13

**Authors:** McKenzie Wallace, Alicia Dessain, Soumya Mikkilineni, Richard D. Hammer

**Affiliations:** Department of Pathology and Anatomical Sciences, University of Missouri, One Hospital Drive, Columbia, MO 65212 USA

**Keywords:** Epstein-Barr virus, EBV-positive mucocutaneous ulcer, Post-transplant lymphoproliferative disorder, Gastrointestinal tract, Liver transplantation

## Abstract

**Background:**

Epstein-Barr virus (EBV)–driven lymphoproliferative disorders (LPDs) are important complications of post-transplant immunosuppression. EBV-positive mucocutaneous ulcer (EBV-MCU) is typically a localized, solitary, sharply circumscribed ulcer with an indolent clinical course, whereas post-transplant lymphoproliferative disorder (PTLD) may be multifocal, disseminated, and life-threatening; however, these entities can overlap histologically on limited gastrointestinal biopsies.

**Case Presentation:**

We report a 60-year-old woman with autoimmune hepatitis/primary sclerosing cholangitis (PSC) status-post liver transplantation (2017; re-transplant 2019 for recurrent PSC) who presented with diarrheal illness and subsequently developed gastrointestinal bleeding. Imaging showed enterocolitis with periportal/mesenteric lymphadenopathy, and endoscopy demonstrated severe colitis with multiple superficial ulcers, without a mass lesion. Biopsies revealed severe active ileocolitis with ulceration and an atypical EBV-positive B cell proliferation (CD20/PAX5+, MUM1+, variably CD30+, EBV-LMP1+; polytypic light chains). The differential diagnosis included EBV-MCU and PTLD -  although the superficial ulcerative pattern and absence of a mass initially raised consideration of EBV-MCU, the presence of multiple ulcers and lack of classic EBV-MCU histologic features favored EBV-positive polymorphic PTLD.

**Management and Results:**

Later, a PET-CT demonstrated extensive FDG-avid lymphadenopathy above and below the diaphragm with splenic and adrenal involvement, supporting disseminated EBV-positive PTLD and prompting rituximab-based chemotherapy.

**Conclusion:**

This case highlights the practical challenges of classifying EBV-positive ulcerative gastrointestinal lesions on small biopsies and underscores the need for radiologic and clinical correlation and close follow-up when EBV-MCU and PTLD are both plausible.

## Introduction

Epstein-Barr virus (EBV) is a ubiquitous gamma-herpesvirus that establishes lifelong latency in B cells. While most primary infections are asymptomatic or manifest as self-limited mononucleosis, EBV can drive lymphoproliferation in immunosuppressed individuals, leading to a spectrum of EBV-associated lymphoproliferative disorders (EBV-LPDs), particularly in solid organ and hematopoietic stem cell transplant recipients [1, 2]. Two important entities at opposite ends of this spectrum are EBV-positive mucocutaneous ulcer (EBV-MCU) and post-transplant lymphoproliferative disorder (PTLD).

EBV-MCU is a recently recognized, indolent B cell LPD that presents as a sharply circumscribed, usually solitary ulcer of the skin, oropharyngeal mucosa, or gastrointestinal tract, typically in the setting of iatrogenic immunosuppression [2, 3, 5, 9]. Histologically, EBV-MCU is characterized by a polymorphous inflammatory infiltrate with scattered large EBV-positive B cells or Hodgkin/Reed-Sternberg-like cells, classically accentuated superficially within the ulcer, associated with a deeper reactive T cell–rich rim [3–5]. This characteristic architecture, together with a solitary circumscribed clinical lesion and lack of systemic disease, helps distinguish EBV-MCU from more aggressive EBV-positive lymphomas and LPDs. Also in contrast to disseminated lymphomas, EBV-MCU frequently resolves with conservative reduction of immunosuppression alone, with reported complete remission rates exceeding 60% and rare progression to systemic disease [2–5].

In contrast, PTLD encompasses a heterogeneous group of lymphoid proliferations ranging from polyclonal hyperplasias to monomorphic B cell lymphomas, most commonly diffuse large B cell lymphoma. PTLD is among the most frequent post-transplant malignancies involving the gastrointestinal tract and often presents with systemic symptoms, mass lesions, and extensive extranodal disease [6–8]. Its prognosis is significantly more guarded, with fewer than 50% of cases responding to reduction of immunosuppression alone and many requiring anti-CD20 therapy and/or chemotherapy [6–8].

Because both entities may present as EBV-positive ulcerative gastrointestinal lesions with overlapping histologic features, distinguishing EBV-MCU from gastrointestinal PTLD can be diagnostically challenging, yet clinically critical given their markedly different prognoses and management. We report a diagnostically challenging case of EBV-positive polymorphic PTLD involving the colon that, upon initial limited clinical workup and small mucosal biopsy sampling, mimicked EBV-MCU.

## Clinical history

A 60-year-old woman with autoimmune hepatitis and primary sclerosing cholangitis underwent orthotopic liver transplantation in 2017 and re-transplantation in 2019 for recurrent primary sclerosing cholangitis. Maintenance immunosuppression included tacrolimus and low-dose corticosteroids. She presented with 1 week of diarrhea, nausea, cough, and congestion, and developed hematochezia with hypotension requiring transfusion. CT angiography demonstrated diffuse enterocolitis and multiple enlarged periportal and mesenteric lymph nodes without a discrete gastrointestinal mass. Colonoscopy showed severe colitis with multiple ulcerations involving terminal ileum, cecum, and transverse colon; no mass lesion was identified.

## Materials and methods

Endoscopic biopsies from terminal ileum and colon were fixed in formalin and routinely processed. Hematoxylin and eosin (H&E)–stained sections were reviewed. Immunohistochemistry included CD3, CD20, PAX5, MUM1, CD30, CD15, CD138, Ki-67, and HSV. EBV was assessed by LMP1 immunostains. Kappa and lambda light-chain in situ hybridization was performed. All immunostains and special stains demonstrated appropriately staining controls. Immunoglobulin gene rearrangement testing was performed at a reference laboratory (Mayo Clinic Laboratories) using a laboratory-validated assay, and results were interpreted in the context of the case findings.

## Results

**Fig. 1:**
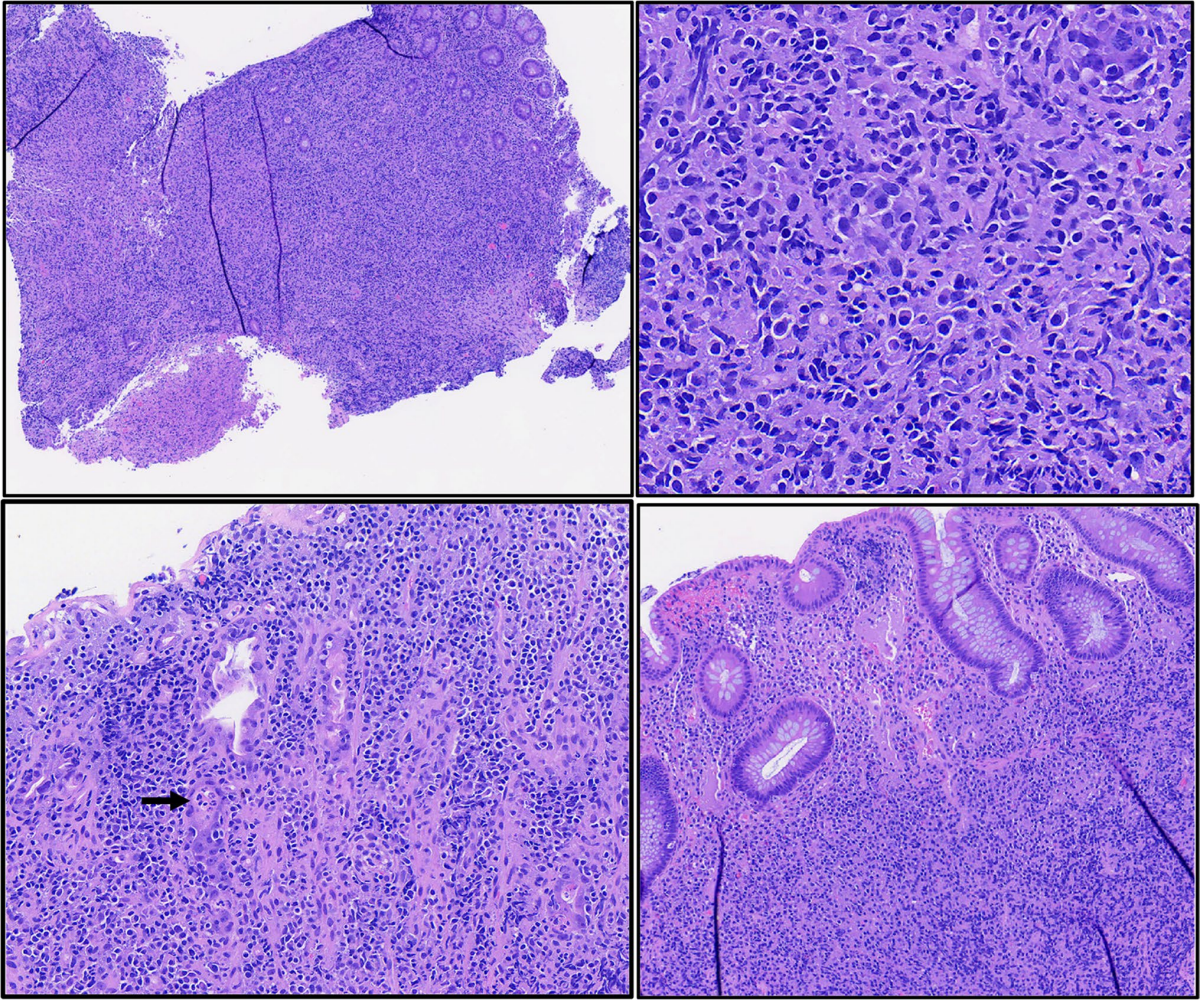
H&E histopathology of colonic biopsy demonstrating polymorphous atypical lymphoid infiltrate in a background of active colitis. Near complete effacement of normal colonic mucosa by an atypical polymorphous lymphoid proliferation (top left). Higher power view, demonstrating polymorphous infiltrate of small mature lymphocytes and occasional plasmacytoid lymphocytes, admixed with intermediate-sized lymphoid cells with hyperchromatic, round to ovoid nuclei and moderate eosinophilic cytoplasm (top right). Background colon exhibited active colitis with cryptitis and crypt abscesses (→) (bottom left and right)

**Fig. 2:**
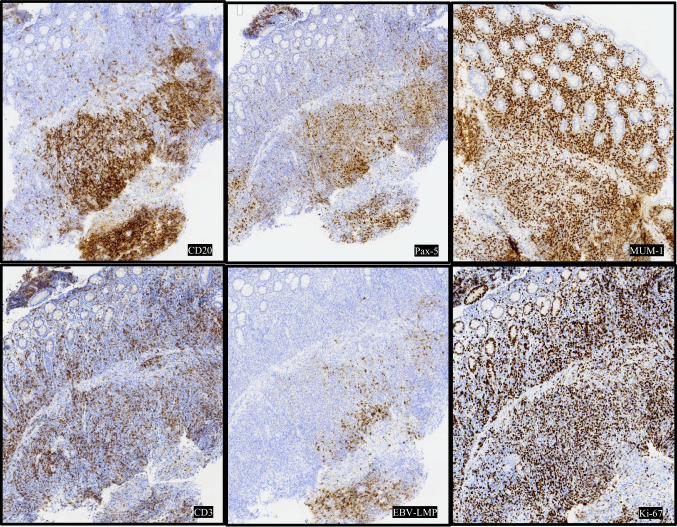
Immunophenotype of the atypical lymphoid proliferation. Immunohistochemical stains for CD20 (top left), PAX5 (top center), MUM1 (top right), CD3 (bottom left), EBV-LMP (bottom center), and Ki-67 (bottom right). The atypical lymphoid cells are positive for CD20, PAX5, MUM1, and EBV-LMP, and (not shown) are variably positive for CD30. CD3 highlights background T cells but is negative in the atypical lymphoid proliferation. The atypical lymphoid cells are also negative for (not shown) CD138, CD15, and HSV. Ki-67 proliferation index is high, highlighting 40–50% of the atypical lymphoid cells

Biopsies demonstrated severe active ileitis and colitis with cryptitis, crypt abscesses, and multifocal ulceration (Fig. 1). Within ulcer bases and adjacent lamina propria/submucosa, there was a polymorphous inflammatory infiltrate containing scattered intermediate-to-large atypical lymphoid cells (Fig. 1). No geographic necrosis and no atypical mitotic figures were identified. The atypical cells were CD20+ and PAX5+, with MUM1 positivity and variable CD30 expression; they were negative for CD3, CD138, and CD15 (Fig. 2). HSV was negative, and GMS showed no fungal elements. Kappa and lambda in situ hybridization demonstrated polytypic light chain expression. EBV-LMP1 immunostain demonstrated numerous EBV-positive cells (Fig. 2). Ki-67 proliferation index was increased within the atypical population (approximately 40–50%). Immunoglobulin gene rearrangement testing was equivocal. The findings on these limited samples were interpreted as an EBV-positive B cell lymphoproliferative disorder, though further classification proved challenging. The superficial ulcerative distribution and lack of a mass lesion raised consideration of EBV-MCU; however, the presence of multiple ulcers and the absence of classic histologic zonation (superficial EBV-positive B cells and a deep T cell–rich rim) were not supportive. Therefore, polymorphic PTLD remained the strongest diagnostic consideration. A conservative management approach with reduction of immunosuppression was initiated, and close clinical follow-up was recommended.

Following the biopsy, transfer to an advanced facility with specialized care for transplant recipients was advised. There, PET-CT revealed markedly FDG-avid, extensive lymphadenopathy above and below the diaphragm, markedly FDG-avid splenomegaly, bilateral adrenal lesions, and cecal/proximal ascending colon wall thickening. These findings supported disseminated EBV-positive PTLD rather than EBV-MCU (Table 1). Combination chemotherapy plus rituximab was initiated. Unfortunately, information regarding additional follow-up is limited due to transfer of care to the outside facility.

**Table 1:** Practical features supporting EBV‑MCU versus PTLD in ulcerative gastrointestinal biopsies

Feature	EBV‑MCU (typical)	PTLD (typical)
Clinical distribution	Localized, sharply circumscribed ulcer; limited sites	Often multifocal/disseminated nodal and extranodal disease
Endoscopy/imaging	Single ulceration without mass; limited lymphadenopathy and no systemic disease	Often multifocal, mass lesions may occur; bulky or widespread lymphadenopathy
Histology on biopsy	Polymorphous infiltrate with superficial EBV+ immunoblasts/Hodgkin-like cells and a deep T cell–rich rim	Variable; may be polymorphic or monomorphic; destructive infiltrates may be deeper
Clonality	May be polyclonal or show restricted clonality	More often clonal (especially monomorphic PTLD), though may be equivocal
Clinical course	Often regresses with reduced immunosuppression	Frequently persists/progresses; may require rituximab ± chemotherapy
Staging (PET‑CT)	When present, uptake generally confined to the lesion; data limited	FDG‑avid disease at multiple nodal/extranodal sites common

## Discussion

This case illustrates a common real-world dilemma; an EBV-positive ulcerative gastrointestinal lesion in a transplant recipient may be difficult to classify on small biopsies, particularly when staging information is incomplete. EBV-MCU and PTLD differ significantly in terms of prognosis, clinical behavior, and treatment. In this case, EBV-MCU was a reasonable differential diagnostic consideration because the biopsy showed an ulcer-associated EBV-positive B cell proliferation with a polymorphous inflammatory background [2–5]. However, important features argued against EBV-MCU and favored EBV-positive polymorphic PTLD even before PET-CT staging: multiple ulcers rather than a solitary sharply circumscribed ulcer, absence of classic histologic features of EBV-MCU (zonation with superficial EBV-positive B cells and a deeper T cell–rich rim), and concurrent periportal/mesenteric lymphadenopathy. Subsequent PET-CT demonstrating extensive FDG-avid lymphadenopathy with splenic and adrenal involvement supported disseminated EBV-positive PTLD [10, 11] and prompted rituximab-based chemotherapy, consistent with established management approaches for PTLD [7, 8].

Limitations of this report include the small biopsy sample, equivocal immunoglobulin gene rearrangement testing, lack of complete outside follow‑up after transfer, and limited ability to assess the full anatomic extent and architecture of disease from mucosal biopsies alone. Furthermore, we would like to note that while the LMP1 immunostain was useful in detecting EBV in our case, EBER in situ hybridization (ISH) is the preferred test as it is the most sensitive method for detecting EBV in tissue sections where EBV-positive LPD is suspected. LMP1 immunohistochemistry can be useful in practice settings with limited access to EBER-ISH, but it may be less sensitive and should be interpreted in the context of morphology and other findings. In these situations, if LMP1 is negative on initial evaluation, EBER-ISH should ultimately be obtained if possible.

This case highlights the need for caution when considering the diagnosis of EBV-MCU on a limited biopsy when systemic disease is clinically plausible. In these scenarios, staging and longitudinal follow-up are essential and descriptive diagnoses (e.g., “EBV‑positive B‑cell LPD, EBV‑MCU versus PTLD”) may be the most honest and actionable approach. For practicing hematopathologists, this case highlights the importance of integrating endoscopic distribution, radiology (including PET-CT when indicated), and clinical trajectory alongside morphology and immunophenotype when rendering and communicating a diagnosis in the EBV-LPD spectrum.

## Data Availability

No datasets were generated or analysed during the current study.
